# Exploring predictors of low nutritional literacy in maintenance hemodialysis patients: a machine learning and network analysis approach

**DOI:** 10.3389/fpubh.2026.1862524

**Published:** 2026-06-10

**Authors:** Ling Yao, Yanling Liu, Xufeng Tan, Sisi Chen, Yanchun Fang

**Affiliations:** 1 School of Nursing, University of South China, Hengyang, China; 2 The First Affiliated Hospital of Shaoyang University, Shaoyang, China; 3 Department of Public Health, Central Hospital of Shaoyang, Shaoyang, China

**Keywords:** machine learning, maintenance hemodialysis patients, network analysis, nutritional literacy, predictors

## Abstract

**Background:**

Patients undergoing maintenance hemodialysis (MHD) commonly experience substantial self-care challenges due to the complexity and highly restrictive nature of their treatment regimens. Nutritional literacy is a critical determinant of effective self-management in this population. However, the main predictors of low nutritional literacy and their relationships remain unclear, limiting early targeted interventions. This study aimed to identify important predictors of low nutritional literacy in MHD patients and to explore how these predictors relate to each other.

**Methods:**

This study included 712 patients on maintenance hemodialysis (MHD). Latent profile analysis was used to identify individuals with low nutritional literacy, followed by machine learning to screen for key predictors. SHAP analysis was employed to visualize the importance of these predictors. Finally, network analysis was used to investigate the underlying relationships among the key predictors.

**Results:**

The LightGBM model demonstrated the best predictive performance, with an AUC of 0.817 and an F1-score of 0.738. The LightGBM model identified nine key predictors: social support, depression, anxiety, self-efficacy, educational level, dialysis frequency, age, living arrangement (living with a spouse and children), and place of residence. Among these, social support had the highest SHAP value. Network analysis revealed that self-efficacy served as the central predictor(rs = 0.56), showing a significant positive correlation with social support (EW = 0.148) and significant negative correlations with depression and anxiety(EW = -0.209, EW = -0.158). The stability and accuracy analyses of the network demonstrated good overall stability, with a correlation stability (CS) coefficient for strength centrality of 0.67. In addition, the Bootstrap 95% confidence intervals were within an acceptable range.

**Conclusion:**

In this study, the LightGBM model was used to identify nine key predictors of low nutritional literacy in MHD patients. Self-efficacy was confirmed as a core node in the predictor network for low nutritional literacy in this population. These findings provide reliable empirical evidence for the early clinical identification of MHD patients at high risk for low nutritional literacy. Furthermore, using self-efficacy as a core intervention target, healthcare professionals can implement targeted interventions for high-risk populations to effectively improve their nutritional literacy. This study offers important practical guidance for optimizing nutritional literacy and intervention strategies for MHD patients.

## 1 Introduction

The global burden of chronic kidney disease (CKD) is steadily increasing. It is estimated that by 2030, approximately 5.4 million individuals will require some form of renal replacement therapy due to kidney failure (1). Maintenance hemodialysis (MHD) is the most common form of renal replacement therapy for patients with kidney disease, accounting for 89% of all dialysis treatments (2). Due to the complexity and highly restrictive nature of treatment regimens, patients undergoing MHD commonly experience significant self-care challenges (3), among which dietary non-adherence is particularly prominent, with reported prevalence ranging from 41.1 to 98.3% (4). This includes difficulties in correctly identifying dietary restrictions such as sodium, phosphorus, and potassium intake, as well as poor dietary self-discipline in daily life. These maladaptive dietary behaviors increase the risk of various serious health complications in MHD patients, including cardiovascular events, renal osteodystrophy, frequent hospitalizations, and increased mortality (5–7). Previous studies have demonstrated a significant positive correlation between nutritional literacy and dietary adherence (3). “Nutritional literacy” is widely used in nutrition and dietary practice research and refers to an individual’s ability to access and understand nutrition-related information and make appropriate dietary decisions based on that knowledge. A study indicated that MHD patients experience greater difficulty in accessing, understanding, and processing nutritional information compared with general health information (3). Compared with general health literacy, nutritional literacy is more closely aligned with the core health needs of MHD patients. Growing attention has been paid to assessing and intervening in nutritional literacy in MHD patients. However, current studies have treated this population as a homogeneous group. They did not account for the large individual differences in demographics and socioeconomic background. Consequently, few studies have specifically focused on MHD patients with low nutritional literacy. MHD patients with low nutritional literacy often show poor control of high-salt, high-phosphorus, and high-potassium diets and difficulty managing fluid intake (3, 8). Moreover, these patients are less compliant with nutrition guidance and intervention plans from healthcare providers (9). This low compliance ultimately weakens the overall effectiveness of nutritional care in dialysis treatment. Therefore, it is essential to identify specific subgroups of MHD patients with poor nutritional literacy, strengthen their knowledge of nutrition and diet, enhance their ability to apply and manage this knowledge, and improve their nutrition-related behaviors.

However, the specific distribution characteristics of low nutritional literacy among MHD patients and the prioritization of key predictive factors remain unclear. In addition, evidence regarding how these predictors co-occur and mutually reinforce one another is lacking. This knowledge gap substantially hinders the accurate identification of high-risk populations and the precise development of interventions in clinical practice, thereby limiting the ability to proactively address the fundamental issue of insufficient nutritional awareness among MHD patients. Therefore, this study aims to focus on low nutritional literacy among MHD patients, to systematically identify its predictive factors, determine their relative importance, and pinpoint precise intervention targets. Machine learning methods were employed to identify key predictive factors, while SHAP analysis was used to visualize the relative importance and prioritization of these predictors. Furthermore, network analysis was conducted to elucidate the interrelationships and synergistic patterns among variables. These complementary methods provide scientific evidence for optimizing nutrition literacy strategies, improving nutrition literacy levels, and reducing the risk of adverse health outcomes in clinical practice among MHD patients.

**Figure 1:**
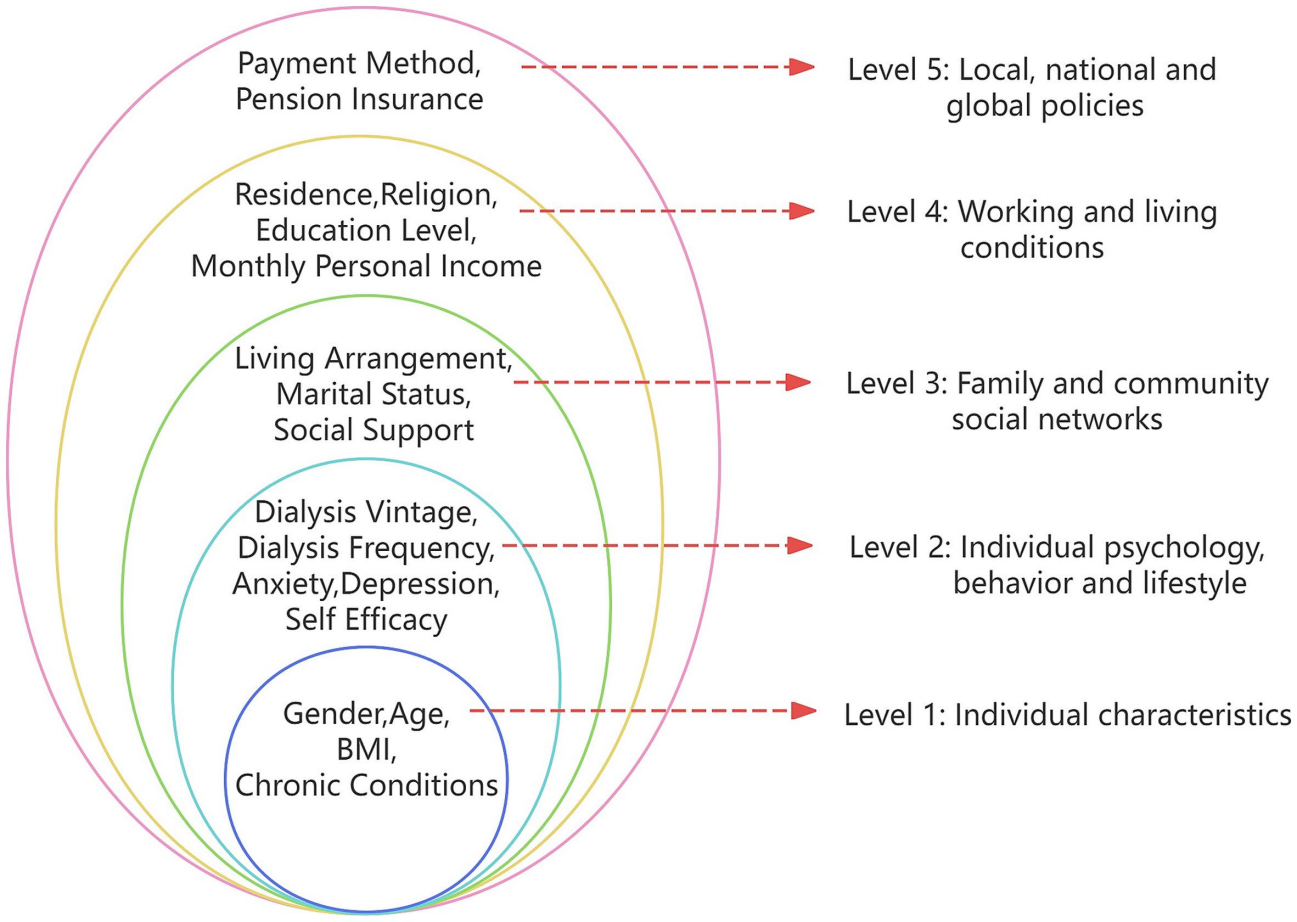
Architecture diagram of predictors of health ecology model theory.

Based on existing literature and clinical expertise, a total of 18 predictive variables were selected for inclusion in this study. Meanwhile, Guided by the social-ecological model of health, this study performed standardized stratification of predictors of nutritional literacy in MHD patients across five levels: individual characteristics; individual psychological, behavioral, and lifestyle factors; family and community interpersonal networks; living and working conditions; and local, national, and global policy contexts. At the individual characteristics level, variables included sex, age, and body mass index (BMI). The psychological, behavioral, and lifestyle level encompassed factors such as anxiety, depression, self-efficacy, and dialysis duration. The family and community network level included marital status,living arrangement, and social support. The living and working conditions level represented educational attainment, religious beliefs, and monthly income. The outermost level—local, national, and global policy context—referred to relevant regulations and policies, including payment mechanisms such as the availability of medical insurance and pension coverage (See Figure 1).

## 2 Materials and methods

### 2.1 Participants and procedure

The research team conducted a cross-sectional, offline, and anonymous survey using purposive sampling among MHD patients from four hospitals in Shaoyang, Hunan Province, and Guilin, Guangxi Zhuang Autonomous Region, between October 2023 and December 2024. The inclusion criteria were as follows: ① receiving regular hemodialysis for ≥3 months; ② aged 18 years or older; ③ able to communicate effectively; ④ willing to participate in the study and provide written informed consent. The exclusion criteria included: ① presence of severe mental disorders or physical illnesses; ② concurrent peritoneal dialysis; ③ a score ≤60 on the Activities of Daily Living (ADL) scale.

At each study center, uniformly trained researchers screened participants strictly according to the inclusion and exclusion criteria. Prior to the formal survey, a pilot study of 30 MHD patients was conducted to assess the validity, reliability, and feasibility of the survey instruments. Before administering the questionnaire, trained staff explained the study objectives and procedures to patients and their caregivers, and then obtained informed consent. The questionnaire was primarily self-administered. For participants who were unable or unwilling to read or write, the investigator read the questions aloud and recorded their answers exactly as given. Upon completion of the questionnaire, investigators immediately reviewed each item on-site to identify and rectify any omissions or inconsistencies prior to collection.

### 2.2 Measures

#### 2.2.1 Nutritional literacy

Developed by our research team in 2023 based on the Health Literacy Hierarchy Theory and the Knowledge-Belief-Behavior Theory (10), this instrument was designed to assess the nutritional literacy levels of patients on maintenance hemodialysis. It comprised five dimensions: nutritional attitudes, nutritional knowledge, nutritional skills, information interaction, and information evaluation. Participants were asked to rate 25 items on a scale ranging from 1 (strongly disagree) to 5 (strongly agree). The total score ranged from 25 to 125, with higher scores indicating higher levels of nutritional literacy. In this study, Cronbach’s alpha was 0.892.

#### 2.2.2 Predictors

##### 2.2.2.1 General information questionnaire

This questionnaire was developed by the research team based on the health ecology model and included items such as gender, age, educational level, and marital status.

##### 2.2.2.2 Anxiety and depression

The Hospital Anxiety and Depression Scale (HADS) was used to assess the severity of anxiety and depression in patients (11). This scale was developed by Zigmond and Snaith (11) and consists of two dimensions (anxiety and depression) and 14 items. Scores for each subscale range from 0 to 21, with higher scores indicating more severe anxiety or depressive symptoms. The scale demonstrated good internal consistency and reliability; in this study, the overall Cronbach’s *α* coefficient was 0.886, while the Cronbach’s α coefficients for the anxiety and depression dimensions were 0.876 and 0.866, respectively.

##### 2.2.2.3 Social support

The Social Support Rating Scale (SSRS) was used to evaluate the level of social support (12). It comprised three dimensions—objective support, subjective support, and utilization of social support—and included 10 items. The total score was the sum of all item scores, ranging from 12 to 66, with higher scores indicating greater levels of social support. In this study, the Cronbach’s *α* coefficient for the scale was 0.853.

##### 2.2.2.4 Self-efficacy

The Chronic Disease Self-Efficacy Scale assessed patients’ self-efficacy (13). The scale comprised two dimensions—disease management self-efficacy and symptom management self-efficacy—and six items. Each item was rated on a scale of 1 (no confidence at all) to 10 (absolutely confident); The total score ranged from 6 to 60, with higher scores indicating greater self-efficacy. The Cronbach’s *α* coefficient for this scale in this study was 0.926.

### 2.3 Statistical analysis

Statistical analyses were conducted using Mplus 7.0, R version 4.5.1, and R Studio. Based on Curran’s study (14), data are considered to follow a normal distribution if the absolute value of skewness is less than 2 and the absolute value of kurtosis is less than 7. Therefore, continuous variables that follow a normal distribution were summarized using the mean and standard deviation, while those that do not were summarized using the median and interquartile range. Categorical variables were reported as percentages and frequencies.

#### 2.3.1 Latent profile analysis

First, LPA analysis was performed at the dimensional level to identify nutritional literacy patterns across different clusters; the analysis began with a single-class model, and additional classes were iteratively added to identify the most appropriate model. Model fit was evaluated using criteria such as the Akaike Information Criterion (AIC), Bayesian Information Criterion (BIC), sample-size-adjusted BIC (aBIC), entropy, the Bootstrap Likelihood Ratio Test (BLRT), and the LoMendel-Rubin-adjusted Likelihood Ratio Test (LMRT). Lower AIC, BIC, and aBIC values indicated better model fit, while significant BLRT and LMRT results indicated that a k-class model fitted the data more effectively than a (k-1)-class model. Higher entropy values indicated more accurate classification. Additionally, previous methodological recommendations suggested that LPA should ideally be conducted with a sample size exceeding 500 (15), and it was necessary to ensure that the smallest category accounts for at least 5% of the total sample (16).

#### 2.3.2 ML model

The dataset was randomly split into a training set (70%) and a test set (30%), comprising 18 predictor variables used to predict the occurrence of low nutritional literacy. During data preprocessing, multinomial categorical variables (e.g., marital status and living arrangement) were encoded using one-hot encoding. To address the class imbalance in nutritional literacy among MHD patients and to improve model stability and generalizability during training, the Synthetic Minority Over-sampling Technique (SMOTE) was applied to the training set with an oversampling ratio of 0.8. The test set retained the original sample distribution without any resampling procedures, ensuring the objectivity and validity of the model generalization performance evaluation.

Model tuning was performed using 10-fold cross-validation combined with grid search on the training set; the optimal hyperparameters were then applied to the test set for performance evaluation. Eight supervised ML algorithms were implemented: decision trees, random forests, SVM, XGBoost, k-nearest neighbors (KNN), Lasso, logistic regression, and LightGBM. Model performance was evaluated using the following metrics: receiver operating characteristic area under the curve (ROC-AUC), accuracy, precision, recall, and F1 score. The LightGBM model demonstrated the best performance among all models. For the LightGBM model, the hyperparameter search space included a tree depth of 3–6, number of trees ranging from 300 to 800, learning rate tuned on a logarithmic scale between 0.001 and 0.032, subsampling ratio of 0.5–0.8, number of features per tree of 5–12, minimum number of samples per leaf of 50–200, and minimum split gain tuned on a logarithmic scale between 0.01 and 100. A total of five random search iterations were explored. The optimal hyperparameters were ultimately selected based on the ROC-AUC metric, yielding a maximum tree depth of 6, a number of trees of 540, a learning rate of 0.00193, a subsampling ratio of 0.774, a number of features per tree of 7, a minimum number of samples per leaf of 79, and a minimum split gain of 3.73.

Since SHAP values based on cooperative game theory not only help explain model outputs but also assess the importance and direction of features, this study used them to visualize the prioritization of key predictors of nutritional literacy (17). The analysis was conducted using the R packages iml (version 0.11.4), fastshap (version 0.1.1), and shapviz (version 0.10.3). SHAP values were computed using the Shapley sampling algorithm with the number of Monte Carlo simulations set to 10. Global model interpretability was achieved through feature importance ranking, SHAP waterfall plot, and feature dependence plots, all showing the top 15 most important features.

#### 2.3.3 Network analysis

A network model of key predictors associated with nutritional literacy was constructed, in which nodes represented the predictors and edges denoted conditional independence relationships among them. It is recommended that the sample size for network analysis should exceed the total number of parameters, including threshold parameters and pairwise association parameters (18). The number of threshold parameters equaled the number of nodes, and the number of pairwise association parameters is calculated as: total number of nodes × (total number of nodes − 1) / 2. In this study, nine core predictors were included in the network analysis; therefore, the sample size exceeding 45 was required. A mixed graphical model (MGM) was used to construct an undirected weighted network with L1 (LASSO) regularization. The regularization parameter *λ* was automatically selected by the extended Bayesian Information Criterion (EBIC). Positive correlations were represented by blue edges, negative correlations by red edges, and edge thickness indicated the strength of the association. Using a spring-embedded layout, nodes with stronger connections were placed closer to each other at the center of the network. Centrality analysis was performed using two indices: strength and expected influence. Next, network accuracy and stability were further assessed using bootstrap procedures implemented in the R package bootnet (version 1.6). The accuracy of network connections was evaluated by calculating the 95% confidence intervals (CI) for edge weights. Bootstrap resampling was used to assess the correlation stability (CS) coefficient and overall network stability. A CS coefficient greater than 0.50 indicated good stability, with a minimum acceptable threshold of 0.25.

## 3 Results

### 3.1 Participants characteristics

This study distributed 756 questionnaires. We received 712 valid responses, giving a 94.18% effective response rate. Using purposive sampling, this study ultimately enrolled 712 hemodialysis patients from Hunan and Guangxi: 555 from Shaoyang (77.90%) and 157 from Guilin (22.10%). Among them, 429 were male (60.3%) and 283 were female (39.7%). Patients under the age of 60 accounted for 47.1% of the total. Table 1 describes the demographic characteristics of the participants. See Table 1 in the Supplementary materials for the skewness and kurtosis values of the continuous variables.

**Table 1:** Sociodemographic characteristics and variable description of patients undergoing hemodialysis.

Category	Variable	Category	Mean (SD) or *n* (%)
Personal Characteristics	Gender	Male	429 (60.30)
		Female	283 (39.70)
	Age	<60 years	335 (47.10)
		60 ~ 69 years	186 (26.10)
		≥70 years	191 (26.80)
	Presence of other chronic diseases	No	282 (39.60)
		Yes	430 (60.40)
	BMI*	—	22.72 (2.94)
Behavioral, lifestyle, and psychological characteristics	Dialysis duration	<24 months	170 (23.90)
		25 ~ 36 months	117 (16.40)
		>37 months	425 (59.70)
	Dialysis frequency	Once per week	26 (3.60)
		Twice per week	231 (32.40)
		Five times every 2 weeks	185 (26.00)
		Three times per week	270 (37.90)
	Anxiety*	—	8.06 (4.54)
	Depression*	—	8.63 (4.44)
	Self-efficacy*	—	34.54 (12.80)
Family and community social network	Social support*	—	39.36 (10.47)
	Marital status	Married	656 (92.10)
		Unmarried	37 (5.20)
		Divorced	19 (2.70)
	Living arrangement	Living with spouse and children	328 (46.10)
		Living with spouse only	206 (28.90)
		Living with children only	71 (10.00)
		Living alone	72 (10.10)
		Other	35 (4.90)
Living and working conditions	Educational level	Primary school or below	210 (29.50)
		Junior high school	260 (36.50)
		High school/technical secondary school	163 (22.90)
		College degree or above	79 (11.10)
	Monthly income (RMB)	<1,000	298 (41.90)
		1,000 ~ 2,999	196 (27.50)
		3,000 ~ 4,999	158 (22.20)
		≥5,000	60 (8.40)
	Residence	Urban	511 (71.80)
		Rural	201 (28.20)
	Religious belief	No	682 (95.80)
		Yes	30 (4.20)
Local, national, and global policy factors	Endowment insurance	No	363 (51.00)
		Yes	349 (49.00)
	Medical insurance	No	20 (2.80)
		Yes	692 (97.20)

*Indicates that the continuous variable follows a normal distribution as confirmed by tests of skewness and kurtosis, and is presented as the mean and standard deviation; categorical variables are presented as the number of cases (percentage) [*n* (%)]. BMI, Body Mass Index.

### 3.2 Nutritional literacy categories

**Figure 2:**
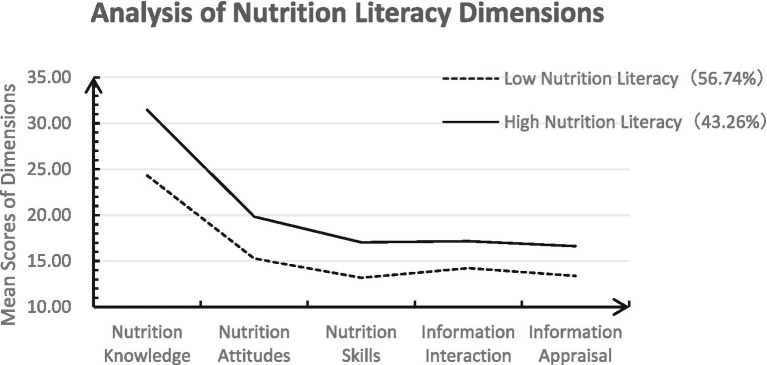
The two profiles of nutritional literacy by LPA.

We fitted latent class models for 1 to 5 classes (see Table 2 for fit indices). AIC and aBIC decreased as the class number increased, while BIC was lowest for the 2-class solution. The 3-class model showed the highest entropy (0.886), indicating the best classification accuracy. The three-class and five-class models each had a class proportion <5%, violating the interpretability criterion recommended by Wendt et al. (16). Although LMR and BLRT were significant for both solutions, we selected the two-class model as optimal after balancing statistical fit and substantive interpretability. Based on the distribution characteristics of the conditional means across dimension for the two classes(see Supplementary material 2), the latent classes were defined as the high nutritional literacy group (*n* = 308, 43.26%) and the low nutritional literacy group (*n* = 404, 56.74%). The total nutritional literacy score was 89.85 (SD = 12.599) for the whole sample, 80.32 (SD = 6.386) for the low group, and 102.35 (SD = 6.158) for the high group (See Figure 2).

**Table 2:** Fitting index and group size of LPA.

Classes	AIC	BIC	aBIC	Entropy	LMR(p)	BLRT(p)	Latent class probability (%)
1	18497.556	18543.237	18511.484				
**2**	**17527.357**	**17600.446**	**17549.642**	**0.873**	**0.0000**	**0.0000**	**56.74/43.26**
3	17508.801	17609.298	17539.443	0.890	0.0135	0.0000	56.60/41.43/1.97
4	17485.288	17613.194	17524.287	0.826	0.1564	0.0000	54.64/1.40/13.20/30.76
5	17456.135	17611.450	17503.492	0.835	0.0315	0.0000	1.83/53.23/13.62/3.37/27.95

Bold entries reflect selected model. AIC, Akaike Information Criteria; BIC, Bayesian Information Criteria; aBIC, Sample size adjusted Bayesian Information Criteria; BLRT, Parametric bootstrapped likelihood ratio test; LMR, Lo–Mendell–Rubin likelihood ratio test.

### 3.3 Analysis of key predictors nutritional literacy

Before training the ML models, we tested the 18 predictors for multicollinearity. All variance inflation factors (VIF) were below 5, indicating acceptable collinearity. Model performance was compared across eight algorithms, including logistic regression, decision tree, random forest (RF), support vector machine (SVM), XGBoost, k-nearest neighbors (KNN), least absolute shrinkage and selection operator (LASSO), and LightGBM. Detailed results are presented in Table 3.

**Table 3:** Performance results of different machine learning models.

Model	Accuracy	Precision	Recall	F1-score	ROC AUC
Decision tree					
Train data	0.674	0.678	0.812	0.739	0.653
Test data	0.660	0.676	0.770	0.720	0.643
Random forest					
Train data	0.791	0.809	0.826	0.818	0.853
Test data	0.698	0.724	0.754	0.739	0.767
Logistic regression					
Train data	0.730	0.808	0.688	0.743	0.797
Test data	0.679	0.768	0.623	0.688	0.747
KNN					
Train data	0.726	0.812	0.674	0.736	0.810
Test data	0.605	0.683	0.566	0.619	0.678
Lasso					
Train data	0.734	0.755	0.787	0.771	0.791
Test data	0.707	0.740	0.746	0.743	0.767
XGBoost					
Train data	0.748	0.840	0.688	0.756	0.824
Test data	0.674	0.745	0.648	0.693	0.764
Lightgbm					
Train data	**0.706**	**0.767**	**0.677**	**0.719**	**0.784**
Test data	**0.716**	**0.775**	**0.705**	**0.738**	**0.817**
SVM					
Train data	0.724	0.770	0.734	0.751	0.793
Test data	0.707	0.757	0.713	0.734	0.768

Bold entries reflect selected model. The F1-score represents the harmonic mean of precision and recall, while ROC-AUC refers to the area under the receiver operating characteristic curve.

**Figure 3:**
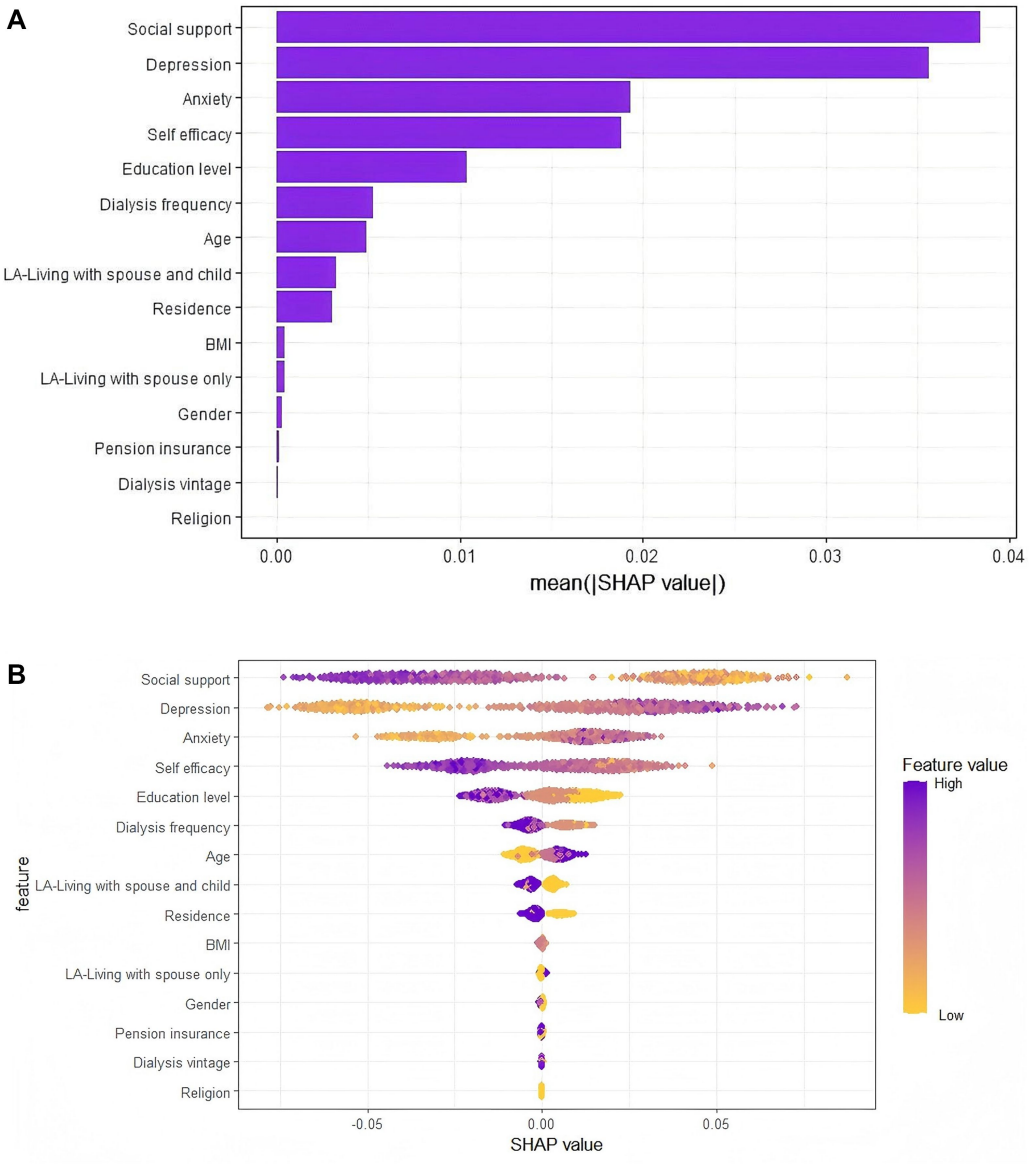
Feature importance and SHAP waterfall plot of the LightGBM model. The figure shows only the top 15 feature factors ranked by importance. **(A)** Bar chart of the average absolute SHAP values for each feature; **(B)** SHAP waterfall plot illustrating the distribution of each feature’s influence on low nutritional literacy. The color gradient in the figure represents the range of feature values (purple indicates high values, yellow indicates low values), and the abbreviation “LA” in the feature labels stands for “living arrangement”.

The LightGBM model showed good performance for predicting low nutritional literacy in hemodialysis patients. On the test set, it achieved an accuracy of 0.716, precision of 0.775, recall of 0.705, F1-score of 0.738, and AUC of 0.817. Based on the mean SHAP values, we ranked the predictors and identified the top 9 as relatively significant predictors (see Figures 3A,B).

**Figure 4:**
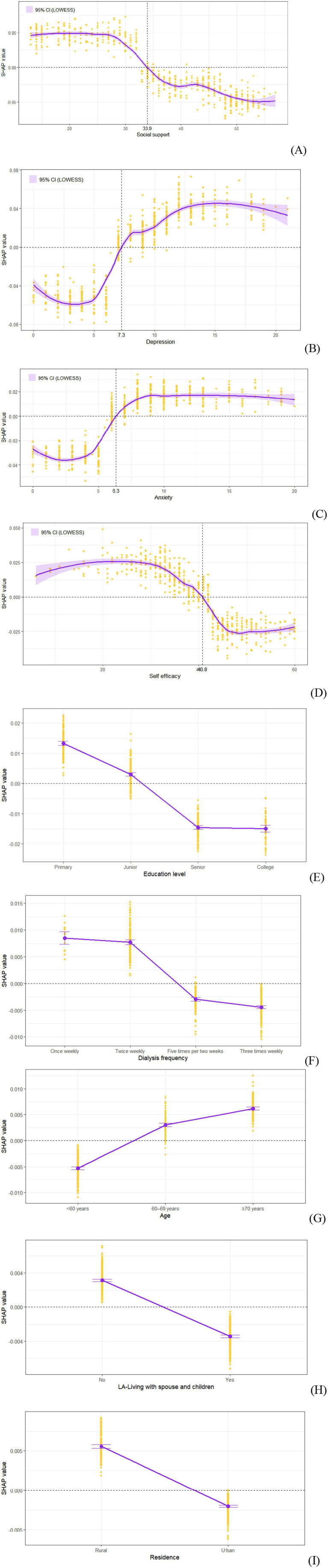
SHAP dependence plots for individual features in the LightGBM model for predicting low nutritional literacy. **(A)** Social support, **(B)** depression, **(C)** anxiety, **(D)** self-efficacy, **(E)** educational level, **(F)** dialysis frequency, **(G)** age, **(H)** living arrangement (living with spouse and children), and **(I)** place of residence. The figure illustrates the relationship between feature values and corresponding SHAP values. For continuous variables, the purple solid line represents the LOWESS fitted curve, and the shaded area indicates the 95% confidence interval (95% CI). For categorical variables, line plots are drawn based on mean SHAP values across categories, with error bars representing the 95% CI. Yellow dots represent individual SHAP values for each sample.

Based on the mean SHAP values, nine factors were identified as key predictors in the model: social support, depression, anxiety, self-efficacy, educational level, dialysis frequency, age, living arrangement (living with spouse or children), and place of residence (Figure 3A). We visualized the marginal effects and influence direction of the key predictors using a SHAP waterfall plot (Figure 3B). SHAP values are referenced to the model’s mean baseline. Positive values indicate increased risk of low nutritional literacy, while negative values suggest a protective effect (reduced risk). We further generated SHAP dependence plots for the key predictors in the LightGBM model (Figure 4), where each point represents one sample in the dataset. For continuous predictors, trends were examined by fitting curves derived from the scatter plots. For categorical predictors, line plots of mean SHAP values were generated, with 95% confidence intervals represented by error bars to illustrate differences between categories. Based on the fitted LOWESS smoothing curves, the effects of social support, depression, anxiety, and self-efficacy on nutritional literacy in MHD patients demonstrated nonlinear patterns. Specifically, when social support scores <33.9, depression scores >7.3, anxiety scores >6.3, or self-efficacy scores <40.8, the corresponding SHAP values were >0, and the risk of low nutritional literacy increased. In addition, lower educational attainment (below junior high school level), dialysis frequency of fewer than two sessions per week, age >70 years, not living with a spouse or children, and rural residence were all associated with a tendency toward low nutritional literacy.

### 3.4 Analysis of the relationships among key predictors of low nutritional literacy

**Figure 5:**
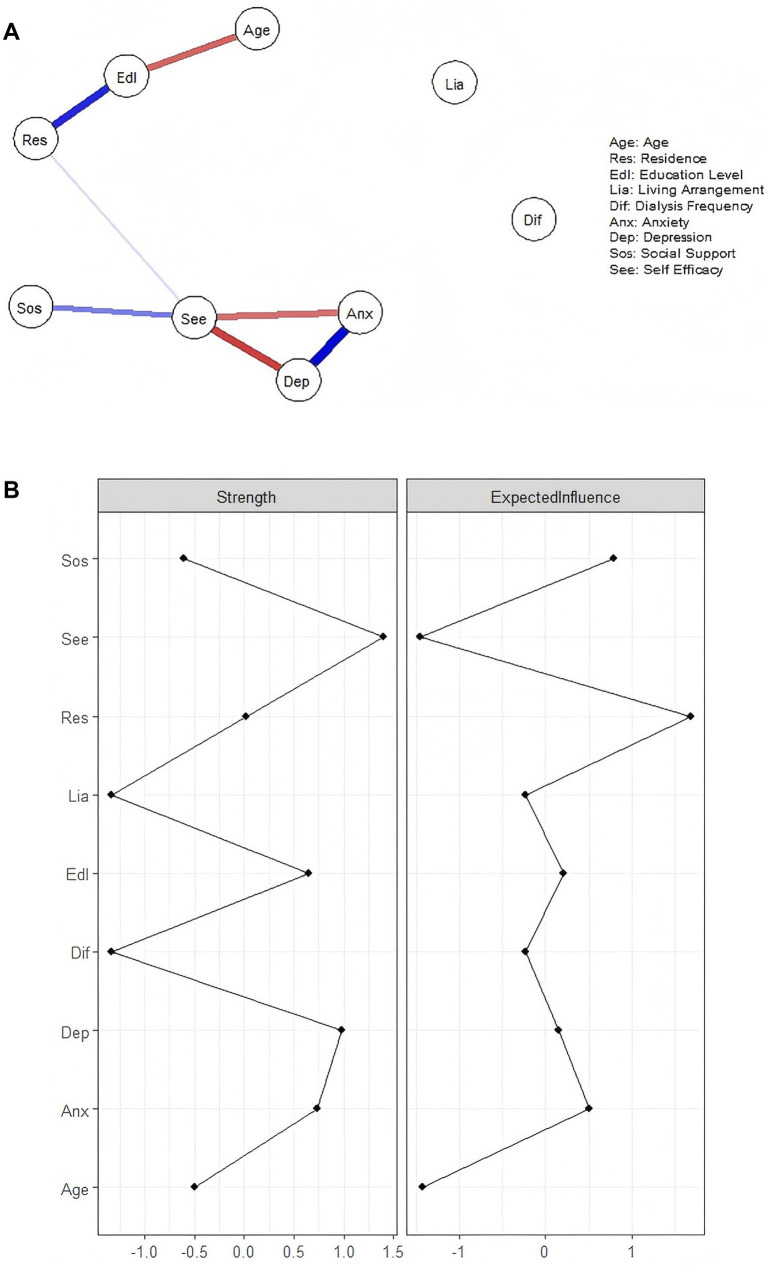
Network analysis of predictors of low nutritional literacy among patients on maintenance hemodialysis: **(A)** Network of 9 key predictors of low nutritional literacy; **(B)** Centrality measures: strength and expected influence of the 9 key predictors.

Network analysis was performed on the top nine predictive factors (Figure 5A). The overall network contained seven edges. Living arrangement and dialysis frequency were isolated nodes and did not show significant associations with the other predictive factors. We calculated strength centrality for the nine predictive factors. The top three factors in terms of strength centrality were self-efficacy (rs = 0.56), depression (rs = 0.47), and anxiety (rs = 0.42). Place of residence had the highest expected influence coefficient (EI = 0.28) (Figure 5B). In this network, the edge between anxiety and depression had the highest weight, with an edge weight of 0.263. Self-efficacy, the core node, was negatively correlated with depression (EW = −0.209) and also with anxiety (EW = −0.158). Social support was positively correlated with self-efficacy (EW = 0.148). The network stability and accuracy analyses indicated that strength centrality had good stability (CS = 0.67 > 0.5), while the stability of expected influence centrality was slightly lower (CS = 0.44 > 0.25). Overall network stability was acceptable. Bootstrap 95% confidence intervals were also within an acceptable range (see Supplementary material 4).

## 4 Discussion

This cross-sectional study used the LightGBM model and network analysis. From 18 candidate variables, we identified predictive factors for low nutritional literacy in MHD patients and explored the associations among these factors. However, studies applying machine learning to predict nutritional literacy outcomes in MHD patients remain limited. Nevertheless, previous studies by Dong et al. and Wang et al. (19, 20). also showed that the LightGBM model had the best predictive performance. It predicted hypotension in dialysis patients (AUC = 0.82) and hypertension in hemodialysis patients (AUC = 0.87). This is similar to our model performance. We could not provide a comprehensive interpretation for all variables. Instead, we focused on the nine core variables that showed the most prominent SHAP values.

### 4.1 Predictors of low nutritional literacy in maintenance hemodialysis patients

This study utilized the LightGBM model and SHAP analysis to identify risk factors for low nutritional literacy among MHD patients and to rank these factors by priority. The top nine predictors were social support, depression, anxiety, self-efficacy, educational level, dialysis frequency, age, living arrangement, and place of residence, consistent with the findings of Tang et al. (21) and Li et al. (21, 22). Furthermore, the results of this study showed that social support had the highest SHAP value in the LightGBM model. This indicates that low levels of social support have a greater predictive effect on low nutritional literacy than other factors. This is likely because nutritional management for MHD patients does not rely solely on individual efforts but requires long-term caregiving support. Nutritional knowledge for MHD patients is more complex than that for other chronic diseases. Furthermore, since most MHD patients are middle-aged or older, they are more likely to experience cognitive decline and memory impairment. Therefore, it is difficult for them to consistently remember complex dietary restrictions and knowledge over the long term. Studies have shown that family-centered social support interventions can better help patients establish evidence-based nutritional decision-making systems (23). A social support network composed of family companionship, peer support, and professional healthcare guidance can provide patients with continuous and diverse sources of nutritional information. Clinically, healthcare providers may leverage social support networks to encourage regular communication between patients, family members, peers, and professionals, while adopting a family-centered intervention perspective. Such strategies may help patients better understand, accept, and apply nutritional knowledge, thereby enhancing confidence and adherence to disease treatment and self-management.

Depression and anxiety are among the leading risk factors for predicting low nutritional literacy in MHD patients, confirming the findings of previous studies (24). Depression may arise from the long treatment course in MHD patients, causing low mood, loss of interest, avoidance behaviors, and reduced social interaction. These factors limit access to nutritional knowledge and information exchange, leading to lower nutritional literacy. In contrast, anxiety is more closely related to acute stress responses, with milder behavioral changes and a smaller negative predictive effect on nutritional literacy. When implementing nutritional literacy interventions for MHD patients in clinical practice, attention should not be limited to dietary knowledge education. The screening and assessment of psychological and emotional status should also be emphasized. Depression and anxiety should be included as routine components of nutritional management evaluation. High-risk patients with depressed mood should be identified early and receive targeted intervention. Psychological counseling and social support can help alleviate their low mood and tendency toward social withdrawal, thereby ensuring effective reception and absorption of nutritional knowledge. At the same time, early management of anxiety should also be emphasized. By using psychological and emotional interventions as a starting point to improve patients’ negative emotions, we can prevent the onset and progression of poor nutritional literacy.

Low levels of self-efficacy also increase the risk of low nutritional literacy among patients with MHD, which is consistent with the findings of several studies (25, 26). Patients with high self-efficacy tend to be more proactive in acquiring knowledge about their condition and developing nutritional skills (27). Therefore, nursing practice should emphasize the critical role of self-efficacy in improving nutritional literacy among patients with MHD. Previous studies have found that self-efficacy moderates the predictive effect of age on nutritional literacy (26). When self-efficacy is low, the negative predictive effect of age on nutritional literacy is more pronounced. This study also shows that age is a predictor of nutritional literacy, with levels decreasing as age increases. This may be due to factors such as physical decline in older adults patients and prolonged dialysis duration, which can lead to reduced self-efficacy, thereby diminishing their confidence and ability in nutritional management. Therefore, older adults MHD patients with low self-efficacy should be identified as a high-risk group requiring priority nutritional literacy interventions in clinical nursing practice. While providing nutritional education, healthcare providers should assess patients’ age-related characteristics and levels of self-efficacy. For older adults dialysis patients with low self-efficacy, progressive nutritional management goals should be established based on their physiological functions and disease progression. Confidence in disease management should be enhanced through positive reinforcement, role models, and feedback on achieving step-by-step goals. This helps patients actively acquire nutritional knowledge and skills, ultimately improving their overall nutritional literacy and long-term dietary adherence.

Educational attainment is also a key factor in predicting low nutritional literacy. Patients with higher education levels can access more nutritional information, understand and apply it better, and communicate and critically evaluate it effectively. These abilities improve their nutritional literacy. This finding is consistent with Liu et al. (28). Furthermore, the results of this study indicate that for MHD patients with an educational level of junior high school or below, higher SHAP values correlate with a greater contribution to predicting low nutritional literacy and a stronger predictive effect on its occurrence. In contrast, for those with an educational level above junior high school, the predictive effect on low nutritional literacy gradually levels off. This suggests that healthcare professionals should develop easy-to-understand nutritional education programs for patients with lower education levels. At the same time, experience sharing and mutual learning should be encouraged among patients and their family members across different educational levels. These approaches can strengthen patients’ nutritional knowledge, improve their practical skills, and ultimately enhance overall nutritional literacy.

Dialysis frequency is also a key factor in predicting nutritional literacy. The results of this study indicate that MHD patients undergoing dialysis more than twice a week are less likely to exhibit low nutritional literacy. This may be because more frequent dialysis sessions increase patient contact with healthcare professionals, thereby providing greater opportunities for face-to-face health education and nutritional guidance. In a qualitative study, patients reported that healthcare professionals provided professional guidance and individualized recommendations during dialysis regarding meal planning, fluid management and restriction, and adherence to other treatment-related restrictions (29). These interventions helped improve patients’ nutritional literacy, reduce the risk of complications, and enhance quality of life. Therefore, for patients with limited opportunities to receive in-hospital guidance from healthcare professionals,an outpatient intervention model can be implemented. At the same time, family members should be encouraged to participate in the patient’s nutritional care throughout the process. Through remote methods such as telephone follow-ups, online education, and home dietary supervision, regular instruction should be provided on specialized topics including dietary management, phosphorus and potassium restriction, and fluid management. Additionally, nutritional and health training should be provided to family members to enable them to assist in monitoring the patient’s daily diet, fluid intake, and dietary restrictions.

This study also found that rural residence and living arrangements are predictors of low nutritional literacy. Consistent with previous research (30, 31), patients living in urban areas may have greater access to structured health promotion activities, more frequent healthcare services, and more reliable sources of nutritional information compared with those in rural areas. Living with a spouse and children significantly reduces the risk of low nutritional literacy. This may be because such living arrangements strengthen family support, and better communication within the family helps patients obtain and use nutritional information. This conclusion has important implications for clinical practice. During patient screening and assessment, healthcare providers should be more sensitive to patients living in rural areas and those lacking family support. This will help identify high-risk populations at an early stage. Furthermore, in developing intervention strategies, efforts should be made to overcome geographical barriers for rural patients. Approaches such as tele-nutrition counseling and training of village doctors can improve service accessibility. At the same time, information dissemination channels should be optimized by using widely accessible media in rural areas, such as the internet and local broadcasting systems. For patients living alone or lacking support from spouses or children, alternative social support systems should be proactively established, such as peer support groups and regular telephone follow-ups. This will fundamentally reduce the risk of low nutritional literacy and lead to better clinical outcomes.

### 4.2 Key intervention targets for low nutritional literacy in maintenance hemodialysis patients

Network analysis was used to explore the relationships among the top nine predictors of low nutritional literacy. The results indicated that self-efficacy serves as a central node and is negatively correlated with anxiety and depression. This is highly consistent with Bandura’s theory of self-efficacy (32). An individual’s belief in their own ability to cope directly affects their emotional experience and behavioral motivation. When self-efficacy is low, patients undergoing long-term disease management and dialysis treatment are more likely to feel powerless and hopeless. These feelings can then trigger negative emotions such as anxiety and depression. Meanwhile, the learned helplessness resulting from long-term disease burden further worsens the vicious cycle between low self-efficacy and negative emotions. Ultimately, this leads to a continued decline in patients’ nutritional literacy. This study also shows that self-efficacy has a stronger negative correlation with depression than with anxiety among MHD patients. A meta-analysis by Huang et al. of 34 studies on self-management interventions for chronic diseases showed that these interventions significantly reduced depressive symptoms but did not significantly improve anxiety symptoms (27). This result suggests that, in clinical practice, the association between self-efficacy and depression may be more responsive to intervention. This finding provides a more precise intervention target for improving patients’ nutritional literacy. When delivering nutritional health education, interventions should not be limited to the mere provision of dietary knowledge. Instead, priority should be given to addressing depressed mood and self-efficacy as core entry points. Improving patients’ psychological status and building confidence in disease management can help alleviate the negative impact of emotional distress on nutritional behaviors. In this way, patients’ nutritional literacy can be effectively enhanced, long-term and standardized self-management can be promoted, and ultimately the overall quality of life of dialysis patients can be improved.

Notably, although self-efficacy and social support were positively correlated in this study, they exhibited distinct functional roles. Social support showed the highest SHAP value, indicating that it is the most powerful predictor for identifying patients with low nutritional literacy, whereas self-efficacy demonstrated the greatest strength centrality in the network analysis, suggesting that it may represent a key intervention target. These findings are consistent with Bandura’s social cognitive theory (33). Social support, as an environmental resource, reinforces individuals’ self-efficacy beliefs through informational interactions and emotional support. Self-efficacy, as a core cognitive mechanism, subsequently drives improvements in health behaviors and outcomes, thereby enhancing patients’ nutritional literacy. Existing studies have shown that educational interventions can significantly improve self-efficacy in MHD patients (34). Such interventions typically include interactive discussions, practical strategies for psychological adjustment (e.g., guides for psychological issues, negative emotion management techniques, and therapeutic communication), as well as content on hemodialysis adaptation, disease management, and self-care (including self-efficacy enhancement and stress management). Qualitative research further confirms that developing self-efficacy skills, along with establishing and maintaining the belief in one’s own efficacy, can lead to its gradual accumulation over time (35). Therefore, in clinical practice, healthcare professionals may implement peer-support interventions across multiple settings while encouraging the involvement of family members. In addition, systematic training programs focusing on coping strategies, disease management, and self-care should be developed for patients. Achievable short-term goals should also be established to encourage patients and their family members to actively participate in the development of care plans. This shared decision-making model between healthcare providers and patients can effectively improve patients’ learning outcomes and self-efficacy (36). It positively guides patients to adopt healthier behaviors and enhances their nutritional literacy.

This study also included two independent nodes: living arrangement and dialysis frequency. The differing patterns observed for living arrangement across the two analytical approaches may be attributed to the use of one-hot encoding in the SHAP-based machine learning analysis. This encoding enabled the model to identify that only cohabitation with a spouse and children had a significant predictive effect on low nutritional literacy. However, when “living arrangement” was included as a multi-class variable in the network analysis, its internal heterogeneity influenced the overall results. Different categories of living arrangements exhibited divergent patterns, and “living arrangement” as a composite variable lost its stable association, becoming an independent node in the network structure. In contrast, the SHAP analysis, through one-hot encoding, allowed for the independent comparison of specific living conditions, capturing the distinct effects of different family structures on nutritional literacy. Patients with higher dialysis frequency have more opportunities to receive health education from healthcare professionals in clinical settings, and dialysis frequency may exert an indirect effect by influencing other mediating factors. Therefore, it was designated as an independent node in the network structure. Methodologically, this finding suggests that future studies of a similar nature could introduce approaches capable of exploring the internal heterogeneity of categorical variables. This would help address the limitations of mixture models and network analysis. For example, association rule analysis of categorical variables could be conducted to visually capture the distribution characteristics of different residential types, dialysis frequencies, and nutritional literacy levels.

However, this study has certain limitations. First, our sampling was limited to two regions—Hunan and Guangxi,which restricts the generalizability and applicability of our findings to other areas. Second, although network analysis was employed, the cross-sectional study design still does not allow for causal inference. Future research could consider using a longitudinal study design to explore causal relationships. Third, the machine learning model used in this study did not include a justification of sample size adequacy. Instead, ten-fold cross-validation and grid search were employed for model development and parameter optimization to enhance model stability and generalizability. Model performance indicators were then used to validate model efficacy. Future studies could use Riley’s sample size formula for prediction models to further optimize sample size planning and improve model reliability (37). Fourth, the nutritional literacy assessment tool was co-developed by our team. Although it has been preliminarily applied in previous studies conducted in Hubei and Nanjing, it has not yet undergone large-scale, multi-center external independent validation. Therefore, its generalizability still needs to be further examined in broader populations. Fifth, this study lacked clinical or laboratory variables directly related to nutritional status. Such variables might act as confounding or mediating factors. Future research is encouraged to integrate multiple dimensions of patient data in the analysis. Sixth, the LightGBM model has not yet been externally validated in an independent cohort.

## 5 Conclusion

In this study, the LightGBM model was used to identify nine key predictors of low nutritional literacy in MHD patients. Self-efficacy was confirmed as a core node in the predictor network for low nutritional literacy in this population. These findings provide reliable empirical evidence for the early clinical identification of MHD patients at high risk for low nutritional literacy. Furthermore, using self-efficacy as a core intervention target, healthcare professionals can implement targeted interventions for high-risk populations to effectively improve their nutritional literacy. This study offers important practical guidance for optimizing nutritional literacy and intervention strategies for MHD patients.

## Data Availability

The data supporting the findings of this study are available upon request from the corresponding author. The data are not publicly available due to privacy or ethical restrictions.
