# Supplementary material for: Exploring predictors of low nutritional literacy in maintenance hemodialysis patients: a machine learning and network analysis approach
Source: Front Public Health. 2026 Jun 10;14:1862524. doi: 10.3389/fpubh.2026.1862524 (PMC13292423; doi:10.3389/fpubh.2026.1862524)

Supplementary Material

# **Supplementary Tables**

**Supplementary Table 1.** Skewness and kurtosis statistics for continuous variables.

**Supplementary Table 2.** Comparison of mean scores across dimensions of nutritional literacy levels.

**Supplementary Table 1.** Skewness and kurtosis statistics for continuous variables.

| Continuous Variables | Skewness | | Kurtosis | |
| --- | --- | --- | --- | --- |
|  | Statistic | Std | Statistic | Std |
| Total population nutritional literacy | 0.145 | 0.092 | -0.997 | 0.183 |
| Low nutritional literacy group | -0.301 | 0.121 | -0.176 | 0.242 |
| High nutritional literacy group | 0.251 | 0.139 | -0.417 | 0.277 |
| BMI | 0.264 | 0.092 | 1.058 | 0.183 |
| Anxiety | 0.514 | 0.092 | 0.059 | 0.183 |
| Depression | 0.234 | 0.092 | -0.25 | 0.183 |
| Social support | -0.155 | 0.092 | -1.274 | 0.183 |
| Self-efficacy | -0.328 | 0.092 | 0.288 | 0.183 |

This table reports skewness and excess kurtosis (excess kurtosis = kurtosis – 3). Std denotes the standard error of the statistic.

**Supplementary Table 2.** Comparison of mean scores across dimensions of nutritional literacy levels.

|  | Nutrition Knowledge | Nutrition Attitudes | Nutrition Skills | Information Interaction | Information Appraisal |
| --- | --- | --- | --- | --- | --- |
| Low Nutrition Literacy（n=404, 56.74%） | 24.321 | 15.268 | 13.184 | 14.236 | 13.391 |
| High Nutrition Literacy（n=308, 43.26%） | 31.460 | 19.836 | 17.030 | 17.162 | 16.620 |

# **Supplementary Figures**

**Supplementary Figure1.** Network stability analysis: correlation stability coefficients for strength and expected influence centrality.

**Supplementary Figure2.**Network accuracy analysis: Bootstrap analysis of edge weights.

**Supplementary Figure1.**


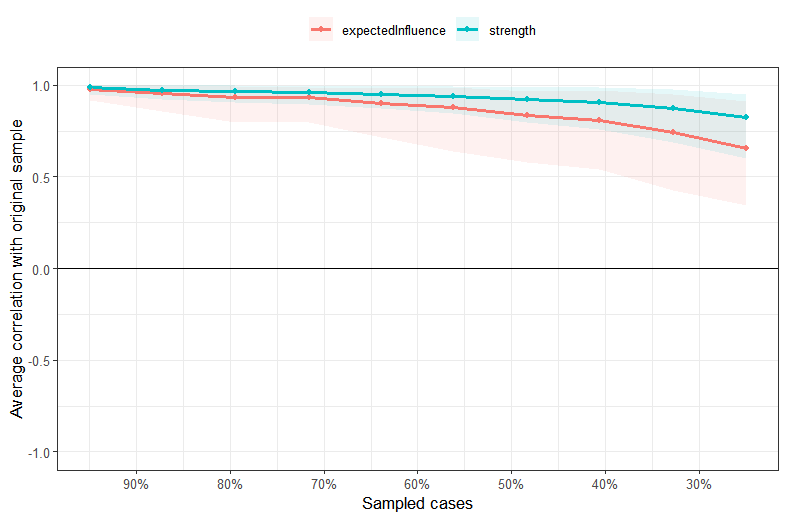


**Supplementary Figure2.**


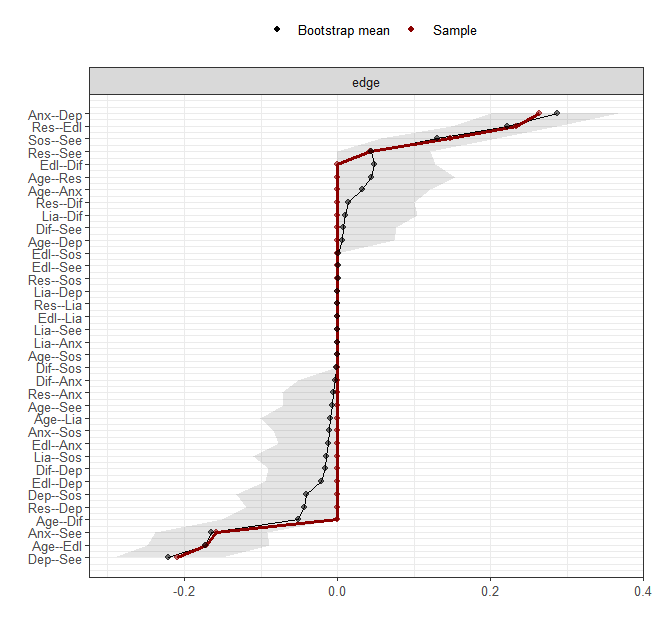

Supplement: Supplementary file 1 [file Data_sheet_1.docx]
